# Lifestyle Planning in the Transition to Retirement

**DOI:** 10.14283/jarlife.2024.4

**Published:** 2024-05-13

**Authors:** S.L. Hutchinson

**Affiliations:** School of Health and Human Performance, Dalhousie University, Halifax, Nova Scotia, Canada

**Keywords:** Challenges, goals, lifestyle, planning, retirement

## Abstract

**Background:**

There is a further need to examine the types of planning people do for their lives in retirement and to examine goals and challenges in relation to planning efforts.

**Objectives:**

This report summarizes highlights from a study that examined retirement planning and explored personal retirement experiences.

**Design:**

An online survey included quantitative and qualitative questions about retirement preparedness and satisfaction and open-ended questions about retirement goals, fears, challenges, and advice.

**Participants:**

Canadians (n = 748) fully or partly retired responded to questions.

**Results:**

Quantitative results determined that while both financial and lifestyle planning were significant predictors of higher perceived preparedness, only lifestyle planning was a significant predictor for perceived satisfaction. Qualitative comments highlighted the importance of goal-setting, including planning for meaningful time use and strategies to address anticipated or existing challenges.

**Conclusions:**

Lifestyle planning is an essential component of planning for the transition to retirement.

## Introduction

**M**ost of us are living longer and healthier. As a result, it matters how we invest our time and other resources as we age. This is particularly so in the transition to retirement. After a lifetime devoted to work or one’s profession and colleagues, now what? Retirement planning workshops and resources abound. Although some may touch on the importance of getting a hobby, very few focus on helping people fully prepare for their lives—and not just their finances—in retirement.

While there has been considerable research on time use, satisfaction, and adjustment following retirement (1-3), there has been less research on what people do to plan or prepare for their retirement lifestyle, including leisure and other forms of time use. Nonetheless, studies consistently report that planning can help people feel like they have some control over the transition to retirement (4-5). Planning enables people to set goals and develop realistic expectations of and feel more prepared for retirement (4).

There is a further need to understand the types of planning people do for their lives in retirement. As it relates to this is there is value in examining people’s goals and challenges in relation to planning efforts. This article summarizes highlights from a Canadian-based study designed to explore retirement goals and experiences in relation to planning for retirement (Hutchinson & Ausman, 2024) (6).

## Study Methods

More information about the study methods is provided in Hutchinson and Ausman (6). In summary, following institutional ethics approval, participants were primarily recruited through Facebook ads targeting profiles with a listed age of 50 years or above and located in Canada. Advertisements directed prospective participants to a website developed for the study where they could find a link to the study’s survey. The survey included demographic questions, followed by both quantitative and qualitative questions.

In brief, statistical analysis was completed using SPSS v.7. Descriptive statistics were used for demographic data, factors including retirement decisions, types of retirement planning, and levels of perceived preparedness and satisfaction. A multinomial regression was conducted to examine the effect of financial and lifestyle planning on perceived preparedness and retirement satisfaction. Content analysis methods were used to analyze the open-ended responses to qualitative questions about retirement goals, fears, challenges, and advice to others contemplating retirement.

## Results

Again, more details about the study’s results are available in Hutchinson and Ausman (6). 748 people from across Canada participated in the study; the majority were women (68%), married/partnered (79.8%), white (97.2%), and fully retired (67.2%).

### Lifestyle Planning

While most participants (68.7%) reported doing some financial planning less than half (47.1%) reported engaging in lifestyle planning. The forms of lifestyle planning reported were diverse, such as reflecting on past/present interests and priorities, reading books, attending workshops, completing self-assessment tools and meeting with a counsellor or life coach. The regression analysis revealed that while both financial planning and lifestyle planning contributed to greater perceptions of preparedness, only lifestyle planning significantly contributed to greater perceived satisfaction. Although all results highlight the importance of lifestyle planning for retirement, the following is a summary of key findings related to retirement goals and challenges. Results related to fears about and advice for others contemplating retirement are provided in the full article (6).

### Retirement goals

When asked to describe goals participants had for themselves in retirement, the most frequently mentioned type of goal was related to participating in meaningful activities (n = 384), such as travel, hobbies, community service and volunteering. Other types of goals were related to health and well-being (n = 222; e.g., physical and mental health, reducing stress, being happy), relationships (n = 190; seeing family and friends, caregiving activities), and time use (n = 129; reducing work or slowing down, staying busy). While there were also comments related to finances (n = 64) and housing (n = 47; e.g., relocating, getting organized or renovating), it is clear that lifestyle factors figured predominantly in study participants’ goals.

### Retirement challenges

When asked what the most challenging part of retirement has been, many challenges were related to time use (n = 180; e.g., not sure what to do with self, boredom) and social isolation (n = 98). Participants also reported financial challenges (n = 84), challenges leaving work behind (n = 55; e.g., not contributing or having no sense of purpose), health and well-being challenges (n = 40; e.g., limited physical activity, changes in sleep), obligations or caregiving responsibilities (n = 29), and challenges related to housing (n = 16; e.g., moving, downsizing, relocation). Importantly, participants described these challenges and strategies they used to deal with them (e.g., returning to work in some capacity, creating routines).

## Discussion

Although only a sample of the study results is provided here, it was compelling that lifestyle factors figured significantly in people’s goals and challenges (as well as fears and advice) related to planning for the transition to retirement. From the statistical results, it is clear that even when people feel financially prepared for retirement, they can still feel unsatisfied if they have not done the work needed to plan for their lives—and not just their finances—in retirement.

Other research has demonstrated the importance of goal setting in the retirement transition process (7). In this study, setting goals for retirement across various life domains (e.g., meaningful activities, health, social connections, travel, personal development, etc.) seemed to be one of the most important planning tasks undertaken by study participants. Participants also described using a myriad of tools or resources to engage in the planning process (e.g., personal research, reflecting on interests and priorities, reading, attending workshops, etc.), which suggests that there is no single best way to engage in planning.

Enjoyable and personally meaningful activities (e.g., leisure) clearly mattered in the study participants’ goals and plans for retirement. It is interesting that leisure and time use have received relatively little attention in the retirement literature (see 8 as an exception). The benefits of leisure for various aspects of health and wellbeing are well established in the literature, including benefits for physical health, social connectedness, and emotional wellbeing (e.g., 9) and these benefits are highly relevant to individuals in the transition to retirement. Further, many of the study participants described goals and plans not just for alleviating boredom or structuring time, but also for meaningful activities and bucket list pursuits. Meaningful time use was also underlying the majority of descriptions of participants’ retirement goals (and advice), including the importance of finding ways to contribute meaningfully to one’s community.

A final important focus of lifestyle planning was to proactively generate strategies or plans for anticipated or existing lifestyle-related challenges, such as health constraints, boredom, or loss of social connections. Trying new things, creating loose routines, returning to some form of work, exploring, and self-discovery—wherein constraints were seen as an opportunity to experiment with new possibilities—are examples of the strategies participants described. The idea that one would plan for and use strategies to optimize remaining resources or overcome barriers is consistent with the concept of ‘life management strategies’ developed by Jopp and Smith (10). It merits further investigation in relation to the retirement transition.

## Implications and Conclusion

In summary, many among the current sample were clearly aware of the benefits of leisure and other meaningful pursuits for optimizing life and well-being in retirement, yet others aren’t. For those who do not value leisure, do not know how to plan for meaningful activities in retirement, or who seem to be struggling psychologically with the transition to retirement (e.g., fear of losing one’s self in the process), then access to relevant education or life coaching seems necessary. In Hutchinson and Ausman (6), a comprehensive list of recommendations for the content of education or coaching based on the study findings is provided.

In conclusion, preparing for new or different ways to use one’s time is an essential component of planning for the retirement transition and later life. Not only is there a significant transition from the routines, requirements, and structures of work to non-work, but beyond this change in daily activities is an equally significant change in how people feel and think about themselves and their lives, which is reflected in how people live their lives. This study’s findings demonstrate Canadian retirees’ perspectives on the need for and value of planning for one’s lifestyle in retirement. While not all study participants engaged in lifestyle planning, those who did were significantly more likely to feel prepared and satisfied with their retirement lives.
